# One Health and Zoonoses: The Evolution of One Health and Incorporation of Zoonoses

**DOI:** 10.5195/cajgh.2015.139

**Published:** 2015-07-23

**Authors:** Govindaraj V. Asokan

**Affiliations:** Public Health Program, College of Health Sciences, University of Bahrain, Manama, Bahrain

**Keywords:** One Health, zoonoses, global health, public health, MeSH

## Abstract

**Introduction:**

Zoonotic disease outbreaks have surged in the last two decades. These include severe acute respiratory syndrome (SARS), Hendra virus, Nipah virus, influenza viruses, Middle East Respiratory Syndrome (MERS) coronavirus, and ebola. One Health is the initiative of an inclusive collaboration linking human, animal, and environmental health. One Health is advocated through an intersectoral coordination to combat zoonoses, and the term has evolved over centuries. The primary aim of this literature review was to examine the change in the definition of the term One Health over time, particuarly following the the introduction of the latest definition in 2007 by the American Medical Association and the American Veterinary Medical Association.

**Methods:**

This review was conducted in four phases. The first phase consisted of a general PubMed search for the phrase “One Health” for every literature published up to December 2014. Then an advanced search was carried out using “One Health” in conjunction with the terms “zoonosis” and “zoonoses” in PubMed for the time period between January 2007 and December 2014. The articles found were then categorized based on the type of journals in which the articles were published. For the second phase, “One Health” was searched as a Medical subject heading (MeSH) term, which is the National Library of Medicine controlled vocabulary thesaurus used for indexing articles. In the third phase, One Health advocate organizations were found using Google search engine. During the final phase, One Health was searched in Google scholar, examined by Google trends, and analyzed by Google ngram.

**Results:**

Before 2007, One Health had many connotations to health in the medical literature with an incomplete adherence to the usage of One Health linking zoonoses. The Google trends analysis shows an overal steady increase of the search of One Health from 2007 to 2014, which is consistent with the findings of articles from Pubmed.

**Discussion:**

Our results indicate that the linkage between the terms One Health and zoonoses started in 2007, which correlates with the joint declaration made by the American Medical Association and the American Veterinary Medical Association in 2007. We suggest creating a MeSH term for One Health in the PubMed database to support more specific research on zoonoses, and exploring the possibility of a patent of the term One Health to support global health and evidence based public health.

An estimated two-thirds of all known infectious diseases and 75% of emerging diseases are zoonoses.^1^ Zoonotic disease outbreaks, often viral, have surged in the last two decades,^2^ highlighting the necessity for a One Health approach. Most emerging viruses were often from the South East Asian Region, such as the severe acute respiratory syndrome (SARS),^3^ Nipah virus,^4^ and the highly pathogenic influenza viruses,^5^ with the exceptions being the Middle East Respiratory Syndrome (MERS)^6^ coronavirus in the Gulf, and the ebola virus in West Africa.^7^ Outbreaks of these diseases have augmented public awareness of the links between wild animals, livestock production, and global public health.^8^

The One Health concept, which originates from ancient civilizations, gained substantial acceptance in the 19^th^ century through infectious disease research^9^ and comparative medicine.^10^ Evolution of the terms related to One Health is summarized in Table 1. In the 19^th^ century, Robert Virchow coined the term “zoonosis” to describe pathogens that are naturally transmitted between vertebrate animals and humans.^11^ Virchow’s contemporary, Sir William Osler, was the first to use the term “one medicine.”^9^ Later, during the 20^th^ century, Calvin Schwabe revived the concept of “one medicine.”^10^ A few years ago, the term “One Health” emerged from the joint efforts of American Veterinary Medical Association (AVMA) and American Medical Association (AMA). By definition, One Health is based on a systems approach, which includes disciplines of human medicine, veterinary medicine, and other related scientific health disciplines, working locally, nationally, and globally, to attain optimal health for people, animals, and our environment.^12^ Currently, One Health aims to develop the capacity and infrastructure to prevent and respond to the rapidly expanding zoonoses through research that is not only focused on the disease but also on the promotion of health at the individual, population, and ecosystem levels.^13^

The concept of One Health strongly emerged in 2007, and has gained acceptance worldwide. To combat the threats of zoonoses, One Health is advocated through intersectoral coordination.^14^–^16^ In 2008, the United Nations established a framework for approaching emerging diseases by establishing intersectoral coordination and communication strategies, which, in turn, enhances surveillance and emergency response of systems at the national, regional, and international levels.^14^ This new approach minimizes the risk and global impact of epidemics and pandemics due to emerging infectious diseases. The Office International des Epizooties (OIE) – World Organization for Animal Health endorsed the One Health approach in 2008, as a collaborative and all-encompassing way to address animal and public health globally.^15^ The Centers for Disease Control and Prevention (CDC) established a One Health office in 2009, and in 2010, the European Union reaffirmed its commitment to operate under One Health umbrella.^16^ One Health also gained international prominence through the coordinated efforts of multidisciplinary professionals, such as physicians, veterinarians, ecologists, etc.

The purposes of this literature review are:

- to identify how One Health has been used recently in the medical literature;
- to identify One Health advocates, such as national and international organizations, and academia, as promoters of One Health research;
- to explore the usage of the term One Health though Google Trends and nGram.

## Methods

This literature review was conducted in four phases. The first phase consisted of a general search in PubMed for the term “One Health” for any literature published up to December 2014. This search returned 1,682 articles, with the oldest article from January 1953. The articles found by the general search term “One Health” were not necessarily linked to zoonoses but had a multitude of meanings, such as One Health district, region authority, system, care, resource, sciences, etc. Literature published beginning in November 2006 was more associated to zoonoses. In order to examine the effect of the 2007 definition of One Health, we excluded articles prior to 2007 with no link to zoonosis. After exclusion of such articles, an advanced search using the term “zoonosis” AND “One Health” returned 193 articles published from January 2007 to December 2014. Similarly, an advanced search for the term “zoonoses” AND “One Health” identified 188 articles from January 2007 to December 2014. Through an advanced search, “zoonosis AND one health” and “zoonoses AND one health” terms were searched for the period between January 2007 and December 2014. The articles found were then categorized based on the type of journals in which the articles were published. For the second phase, “One Health” was searched as a Medical subject heading (MeSH)^17^ term, which is the National Library of Medicine controlled vocabulary thesaurus used for indexing articles. In the third phase, One Health advocate organizations were found using Google search engine. During the final phase, “One Health” was searched in Google scholar and examined by Google trends, which analyzes the number of searches for terms in Google.^18^ Google ngram was then used to to analyze the use of the terms in Google books.^19^

## Results

The search results indicate that, in general, there was a gradual increase in the usage of One Health from the year 2007 onwards, with an accelerated usage of the term in 2013 and 2014. The only exceptions were 2010 and 2012, in which there were slight decreases in the usage of One Health (Table 2).

A discipline based journal categorization was almost the same between the two advanced search terms (Table 3). For the search term “zoonosis” AND “One Health,” 71 articles were from veterinary medical journals, out of which 30 articles were listed from Scientific and Technical Review of the OIE alone; 53 articles were listed by public health journals, 34 articles from medical journals, and 33 articles in the others category, which included basic science journals such as ecology, environmental, and wildlife journals.

The most common keywords associated with “One Health” were “Zoonosis” and “Zoonoses,” and our search strategy was confirmed by the returned results. Our search in the MeSH browser for indexing of the term “One Health” found no results.

### One Health Advocates

Google searches identified many prominent organizations that support One Health, including: WHO, FAO, OIE, CDC, AVMA, AMA, One Health Initiative movement, One Health Sweden, World Veterinary Association, One Health Global Network, One Health Commission, One Health Alliance of South Asia, One World–One Health effort, Ecological Society of America, and the World Bank. The OIE, as a One Health advocate, is the largetst contributor, with 30 articles in Scientific and Technical Review of the Journal OIE.

Some universities which support or offer programs in the area of One Health include: University of Edinburgh, One Health Institute of University of California at Davis, Uppsala University, Swedish National Veterinary Institute, Swedish University of Agricultural Sciences, Linnaeus University, One Health/One Medicine initiative at University of Missouri, University of Minnesota, University of Pennsylvania, Oregon State University, Massey University, University of Guelph, Center for One Health at University of Illinois, North Carolina One Health Collaborative, and University of Florida. A few more nongovernmental organizations, namely Bill and Melinda Gates foundation, are allied to One Health.

### Google trends and ngram analysis

**Figure 1:**
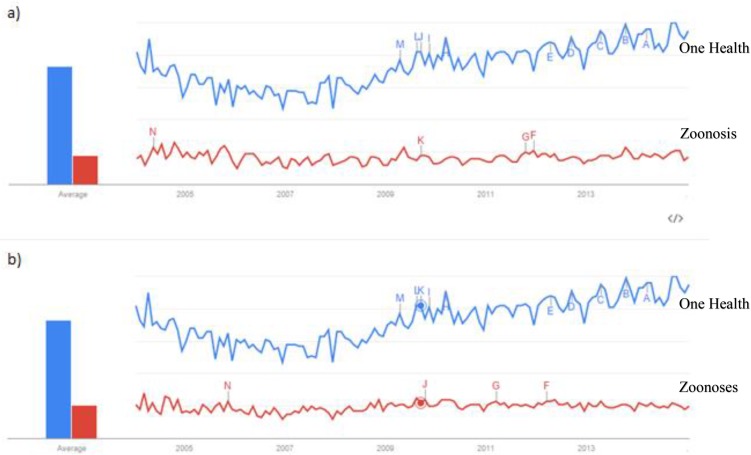
**Google trends analysis of zoonosis/zoonoses and One Health^18^**

The Google trends analysis used data from 2004 to 2014 in order to detect any changes in searches of One Health and zoonoses pre- and post-introduction of the new One Health definition. The Google trends analysis shows a slight decline in searches of One Health from 2004 to 2007 and then an overall steady increase in searches for the term One Health from 2007 to 2014, which is consistent with the findings of articles from Pubmed and the introduction of the new One Health definition (Figure 1). There was no noticeable change in the trend of zoonosis or zoonoses.

**Figure 2:**
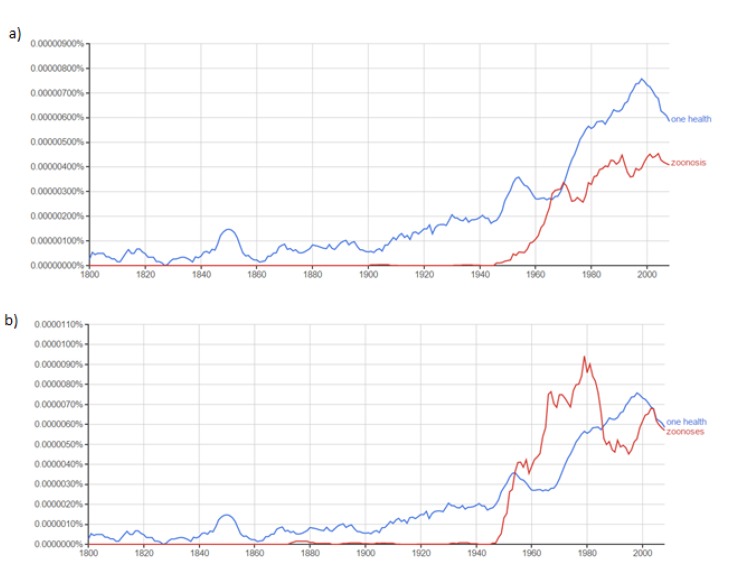
**Ngram analysis of zoonosis/zoonoses and One Health^19^**

A historical ngram analysis of the terms One Health and zoonosis/zoonoses shows an overall increase in the use of One Health and zoonosis/zoonoses, with the greatest increase occurring in the 1940s while tapering off slightly in 2010 (Figure 2).

## Discussion

The prevention of zoonoses that impact public health and animal health requires a sustained collaboration among the stakeholders to promote the One Health approach. Even though One Health has multiple definitions accorded to it by its various advocates, the common theme that has emerged is collaboration across sectors.^20^ The development of Rift Valley fever vaccine by the CDC is one of the noteworthy successes of One Health’s collaborative action.^21^ Such collaboration needs to expand to developing countries at all levels of health care delivery, risk communication, and research. Uninterrupted, accessible, and indispensable information from a reliable source is central to achieving the One Health goals. The most popular, peer reviewed, reliable, and freely available medical and veterinary literature database is PubMed. Physicians, veterinarians, basic science researchers, and eco-health professionals often rely on PubMed for their basic scientific information searchers.

The ngram analysis of the use of One Health over time showed a marked increase roughly around 1945. The 1960s to 1980s mark a particular turn of events in infectious disease epidemiology with the eradication of smallpox, and the decrease of incidence and prevalence of many other diseases, such as measles.^22^ With new research on understanding the etiology of the most recent emerging diseases (e.g. swine flu, SARS, etc.), it has been estimated that roughly 75% of emerging diseases are zoonotic in origin,^22^ which would explain the increase in the study of zoonosis/zoonoses and One Health in the past few decades.

The purpose of this literature review was to identify how the term One Health is used in the medical literature and to identify one health advocates. We were able to identify and list the major One Health advocates through a Google search, where OIE emerges as the most prominent advocate. However, a PubMed search for One Health has shown the zoonoses linkage to the term from the year 2007 only, and this correlates with the joint declaration made on One Health by AVMA and AMA in 2007. Prior to 2007, One Health had many connotations to health in the medical literature. Our results have shown that there is a sustained use of the term One Health from 2007 onwards, with an accelerated use in 2013 and 2014. However, an incomplete adherence to the usage of One Health linking zoonoses in the medical literature is evident, and this practice of poor adherence will potentially impede the implementation of the goals of One Health initiatives. The systems approach of One Health is practical, cost effective, and involves the sustainable, multidisciplinary support of academia and various organizations to undertake more rigorous scientific research for countering the accelerated rise of zoonoses. Such research inquiry on zoonoses compels the correct use of terminologies that are acceptable and comprehensible, without any misuse and misunderstanding in the medical literature. The term One Health suits the current demand of multidisciplinary scientists involved in the development of this term, which evolved with consensus and feedback from multiple disciplines over time. Therefore, we suggest creating a MeSH term for One Health in the PubMed database that could be under the MeSH tree structures of Health [N01. 400] similar to family health, public health, rural health, occupational health, and veteran’s health to support more specific research on zoonoses. Furthermore, exploring the possibility of a comprehensive, definitive definition for the term One Health is a necessity to promote One Health, global health, and evidence based public health.

## Figures and Tables

**Table 1:** Evolution of the term One Health

	Individual/Organization	Experimental Basis	Evolution of the Term
19^th^ Century	Robert Virchow	Experimental studies on Trichinella spiralis, bovine cysticercosis, and bovine tuberculosis	Zoonoses^9^
19^th^ Century	Sir William Osler	Comparative pathology between human and animal diseases	One Medicine^9^
20^th^ Century	Dr. Calvin W. Schwabe	Comparative and collaborative medicine to combat diseases and to ensure food and environment quality	Reintroduced One Medicine^10^
2007	American Medical Association (AMA) and American Veterinary Medical Association (AVMA)	Integrated research, education, and public health prevention, preparedness, and response for achieving improved animal and human health	One Health^10^

**Table 2:** Frequency of articles from January 2007 to December 2014 for advanced search terms in PubMed

Year	Search Terms	

	“zoonosis” and “one health”	“zoonoses” and “one health”
2007	3	3
2008	5	5
2009	12	12
2010	11	11
2011	21	21
2012	13	13
2013	60	56
2014	68	67
Total	193	188

**Table 3:** Stratification of articles by discipline for advanced search terms in PubMed from January 2007 to December 2014

	Search Terms	

	“zoonosis” and “one health”	“zoonoses” and “one health”
Veterinary medicine	71	71
Public health	53	52
Medical	34	32
Health policy	2	2
Other	33	31
Total	193	188

*Note.* Other disciplines include: Ecology health, Wildlife diseases, Nursing, Environmental health, etc.
